# Post renal biopsy complication rate and diagnostic yield comparing hands free (ultrasound-assisted) and ultrasound-guided biopsy techniques of renal allografts and native kidneys

**DOI:** 10.1186/s40064-015-1292-0

**Published:** 2015-09-12

**Authors:** Hatem Ali, Asam Murtaza, John Anderton, Aimun Ahmed

**Affiliations:** Renal Department, Royal Preston Hospitals, Lancashire Teaching Hospitals NHS Foundation Trust, Preston, UK

**Keywords:** Needle guided, Hands free, Renal biopsy, Adequacy, Complications

## Abstract

**Background:**

Real time ultrasound guided percutaneous kidney biopsy has become the standard procedure to assess the pathology of native kidneys and renal transplants. No specific technique has shown to be totally free of post biopsy bleeding complications. Few Studies have looked at the rates of post biopsy bleeding complications comparing different needle size, post biopsy haematoma size, or clinical predictors of the complication rates. In this study we aim to assess safety and adequacy of the real time ultrasound guided biopsy using free hands (ultrasound-assisted) and ultrasound-guided technique.

**Method:**

The results of 527 elective native and kidney transplant biopsy performed as a day case procedure at Lancashire Teaching Hospitals were retrospectively reviewed (499 native and 28 allograft biopsies). Biopsies were grouped into 4 groups according to the technique and the needle size; group 1 (n = 119; performed by free hands-ultrasound assisted- technique using 14G needle) Group 2 (n = 59; performed by free hands-ultrasound-assisted technique using 16G needle), group 3 (n = 195; performed by ultrasound-guided technique using 14G), and group 4 (n = 154; performed by ultrasound-guided technique using 16G). The 4 groups were matched in age, sex, weight, haemoglobin, serum creatinine, INR, PT, and PTT time.

**Results:**

The overall tissue specimen was adequate in 80.45 % of the cases, with no difference between group 1 and 3 (81.5 and 80.52 % respectively, p = 0.82) or between group 2 and 4 (86.44 and 77.3 % respectively, p = 0.13). The overall major complications rate was 2.84 %, with no difference between group 1 and 3 (2.5 and 1 % respectively, p = 0.30) or group 2 and 4 (5 and 4.5 % respectively, p = 0.86). The overall minor complications was 3.7 % with no difference between group 2 and 4 (3.3 and 5.84 % respectively, p = 0.46), however, minor complications were higher in group 1 compared to group 3 (5.8 and 1 % respectively, p = 0.01).There was no difference between using 14G and 16G needle size in terms of tissue adequacy(p = 0.7), major complications (p = 0.2 for drop in Hb >10 g/l, p = 0.08 for blood transfusion, p = 0.35 for embolization) or minor complication items(p = 0.4 for drop in Hb, 10 g/l,p = 0.1 for haematuria, p = 0.7 for hematoma).

**Conclusion:**

When using a 14G needle, there is higher risk of minor complications in the free hands-(ultrasound-assisted) technique compared to the ultrasound-guided technique. There is no difference in the rates of major or minor complications between free hand and needle-guided technique using 16G needles. Both techniques showed adequate tissue sampling.

## Background

Percutaneous needle biopsy of the kidney is one of the most important investigations in assessing renal pathology (Wiczek 1990; Gray et al. 1992; Matas et al. 1985). Technical advances in biopsy procedures have changed from a blind approach to real time ultrasound guided techniques (Donovan et al. 1991). Percutaneous renal biopsies were first performed by Iversen and Brun who used an aspiration biopsy needle (Iversen and Brun 1951). Afterwards, several instruments and methods were introduced. Nowadays, a real time ultrasound guided method has become the standard technique (Cozens et al. 1992; Marwah and Korbet 1996; Burstein et al. 1993; Nass and O’Neill 1999). It has been proved to achieve a better yield and fewer complications than the blind method (Maya et al 2007). Furthermore, a spring loaded automated gun instrument has become the instrument of choice (Burstein et al. 1993; Wiseman et al. 1990; Dowd et al. 1991). As any invasive procedure, renal biopsies carry the risk of several complications, like pain, infection and bleeding. Bleeding complications can present with drop in haemoglobin, peri-renal hematoma, hematuria or formation of arterio-venous fistulas (Meldelssohn and Cole 1995). These complications may require blood transfusion and may lead to loss of the kidney or even death.

Despite the high frequency of performing renal biopsies, the exact rate of bleeding complications is still obscure (Kim et al. 1998). Many data are collected before performing a renal biopsy, trying to predict post biopsy bleeding like blood pressure, haemoglobin, bleeding time, prothrombin and coagulation. However, no study has proved that all these are important to predict occurrence of complications (Burstein et al. 1993; Marvah and Korbet 1996).

Few studies have examined the complication rate of percutaneous ultrasound guided biopsies using spring loaded devices (Bogan et al. 1990; Kolb et al. 1994; Mahoney et al. 1993; Hanas et al. 1992; Mendelssohn and Cole 1995) and most of these studies have focused on this procedure using different needle sizes (Donovan et al. 1991; Kumar et al. 1992; Ogborn and Grimm 1992; Tung et al. 1992). These studies proved that rate of complications and tissue adequacy were higher while using 14G needles than while using 16G needles (Tøndel et al 1988). Several techniques have been used to perform real time ultrasound guided renal biopsies using a spring loaded automated biopsy gun. The most common are needle-guided (ultrasound-guided renal biopsy) and free-hands(ultrasound-assisted renal biopsy) techniques. However, to our knowledge, no study has evaluated the complication rate of real time ultrasound guided biopsies using the two techniques. The aim of our study was to compare the adequacy and complication rate of this procedure comparing hands-free and needle-guided real time ultrasound guided techniques.

## Methods and analysis

Adequacy and complication rates of 527 renal biopsies performed at Lancashire teaching hospitals from January 2010 to end of June 2014 were retrospectively reviewed using a computerised database. All biopsies were performed by seven trained nephrologists.

The technique of renal biopsy (ultrasound-assisted versus ultrasound-guided) was at the discretion of the operator and was based on their past experience performing the kidney biopsy.

Both 14G and 16G sized needles were used with the spring-loaded automated gun (ANGIOTECH Tru-Core II Automatic Biopsy Instrument). The biopsy procedure was performed electively in a day case unit for patients suitable for the procedure on out-patient basis.

Patients signed consent after the procedure had been explained to them. All patients had pre-biopsy renal ultrasound scan and coagulation test that were within normal. Pre-biopsy blood pressure was less than 160/95 for all patients on the day of the biopsy.

Patients who had native kidney biopsy were positioned in prone position with a pillow under the abdomen, with the left kidney as the default site for the biopsy. Patients for transplant kidney biopsy remained in a supine position. The skin was disinfected with chloraprep solution (ChloraPrep is 2 % chlorhexidine combined with 70 % isopropyl alcohol). Blood pressure and oxygen saturation were monitored during the whole procedure.

In ultrasound-assisted renal biopsy technique, the kidney was visualised under ultrasound and the skin over the lower pole of the left kidney was marked. A local anaesthesia was injected via a needle or spinal needle to anesthetise the capsule of the lower pole of the left kidney. A small incision was made in the skin and the spring loaded automated gun was used for all cases and under real-time ultrasound guidance. Number of passes in individual patients in our study were one to three passes.

In ultrasound-guided renal biopsy technique, a guide was braced around the ultrasound probe and under real-time ultrasound guidance, the needle was advanced through the guide to the cortex of the lower pole of the left kidney. Patients were instructed to stay supine for 4–6 h post procedure and serial urine collection of urine were observed for visible haematuria. Vital signs were observed every 30 min for the first 4 h aiming for a blood pressure below 140/90. No post biopsy ultrasound scans were performed to any of the uncomplicated cases to check for peri-renal hematoma.

4 groups were identified; group 1 (ultrasound-assisted renal biopsy technique using 14 G needle), group 2 (ultrasound-assisted renal biopsy technique using 16G needle), group 3 (ultrasound-guided renal biopsy technique using 14G needle), group 4 (ultrasound-guided renal biopsy technique using 16G needle). Clinical characteristics and outcomes were compared between group 1 and 3 and between group 2 and 4. Mann–Whitney test was used for continuous variables and a 2-independent proportion analysis was used for categorical variables. A *p* value <0.05 was statistically significant.

The groups were matched in terms of age, sex, weight, haemoglobin, INR and PT. Major complications were defined as a drop in haemoglobin of equal or more than 10 g/l one week post procedure, requirement of a blood transfusion or embolization. Minor complications were defined as a drop in haemoglobin less than 10 g/l one week post procedure, macroscopic haematuria or peri-renal haematoma. Tissue adequacy was defined as presence of at least 10 glomeruli in the biopsy specimen.

## Results

There were 119 patients (60 males, 59 females) in group 1 and 195 patients (114 males, 81 females) in group 3.

59 patients (35 males, 24 females) were in group 2 compared to 154 patients (87 males, 67 females) in group 4. Average age, weight, haemoglobin, serum creatinine, PT and INR are summarized in Tables 1 and 2.

**Table 1:** Patient characteristicss of group 1 and 3

	Group 1	Group 3	P value
Number	119	195	
Age	59 ± 1	55 ± 1	0.24
Sex (m:f)	60:59	114:81	
Weight	79 ± 9	77.5 ± 8	0.65
Hb	120 ± 9	122 ± 8	0.84
PT	10.7 ± 5	10.7 ± 5	0.33
INR	1 ± 8	1 ± 6	0.09
Creatinine	157 ± 1	122 ± 8	0.08

Data are expressed as median ± standard deviation

**Table 2:** Patient characteristics of group 2 and 4

	Group 2	Group 4	P value
Number	59	154	
Age	63 ± 5	58 ± 3	0.08
Sex (m:f)	35:24	87:67	
Weight	79 ± 7	76. ± 6	0.16
Hb	121 ± 3	120 ± 3	0.57
PT	10.8 ± 4	10.7 ± 1	0.56
INR	1 ± 3	1 ± 3	0.53
Creatinine	140 ± 3	129 ± 3	0.97

Data are expressed as median ± standard deviation

The overall tissue specimen was adequate in 80.45 % of the cases. The tissue specimen was adequate in 97 (81.5 %) and 157 (80.52 %) renal biopsies in group 1 and 3, respectively. There was no difference between group 1 and 3 (p = 0.82). Tissue specimen was adequate in 51 (86.44 %) biopsies in group 2 and 119 (77.3 %) biopsies in group 4. There was no difference between group 2 and 4 (p = 0.13). Overall average number of sampled glomeruli were 20.40. Average number of glomeruli in free-hands technique is 22.72 while average number of glomeruli in needle-guided technique is 18.61.

The overall complications rate was 5.64 %. The overall major complications rate was 2.84 %. Arterio-venous fistulas were not detected in patients who had angiography for embolectomy. There was no significant difference between group 1 and group 3 or between group 2 and group 4 as shown in Tables 3 and 4.

**Table 3:** Major complications in group 1 and 3

	Group 1	Group 3	P value
Number	119	195	
Major complications no. (%)	3 (2.5 %)	2 (1 %)	0.30
Drop in Hb >g/l	2 (1.6 %)	2 (1 %)	0.61
Blood transfusion	0 (0 %)	0 (0 %)	
Embolization	1 (0.9 %)	0 (0 %)	0.19

**Table 4:** Major complications in group 2 and 4

	Group 2	Group 4	P value
Number	59	154	
Major complications no. (%)	3 (5 %)	6 (4.5 %)	0.86
Drop in Hb >g/l	2 (3.3 %)	3 (2.5 %)	0.75
Blood transfusion	0 (0 %)	2 (1.29 %)	0.37
Embolization	1 (1.7 %)	1 (0.6 %)	0.47

The overall minor complications rate was 3.7 %. There was no difference between group 2 and 4, however, minor complications were higher in group 1 compared to group 3 as shown in Tables 5 and 6.

**Table 5:** Minor complications in group 1 and 3

	Group1	Group 3	P value
Number	119	195	
Minor complications no. (%)	7 (5.8 %)	2 (1 %)	0.01
Drop in Hb <10 g/l	5 (4.2 %)	2 (1 %)	0.06
Hematuria	2 (1.8 %)	0 (0 %)	0.4
Hematoma	1 (0.9 %)	0 (0 %)	0.19

**Table 6:** Minor complications in group 2 and 4

	Group 2	Group 4	P value
number	59	154	
Minor complications No. (%)	2 (3.3 %)	9 (5.84 %)	0.46
Drop in Hb <10 g/l	1 (1.7 %)	6 (3.89 %)	0.42
Hematuria	1 (1.7 %)	2 (1.29 %)	0.82
Hematoma	0 (0 %)	1 (0.6 %)	0.53

Also, our data did not show any difference between 14G and 16G needles in terms of major and minor complications as shown in Tables 7 and 8.

**Table 7:** Major complications between 14G and 16G needle

	14G	16G	P value
Number of patients	314	213	
Drop of Hb >10 g/l: No. (%)	4 (1.2 %)	6 (2.8 %)	0.2
Blood transfusion	0	2 (0.9 %)	0.08
Embolization	1 (0.31 %)	2 (0.9 %)	0.35

**Table 8:** Minor complications between 14G and 16Gneedle

	14G	16G	P value
Number of patients	314	213	
Drop of Hb <10 g/l: No. (%)	7 (2.8 %)	7 (3.2 %)	0.4
Hematuria	1 (0.3 %)	3 (1.4 %)	0.1
Hematoma	1 (0.3 %)	1 (0.4 %)	0.7

254 renal biopsies (80.8 %) showed adequate tissue sampling using 14G needle. 0.170 renal biopsies (79.8 %) showed adequate tissue sampling using 16G needle. There was no difference in terms of tissue adequacy between 14G and 16G needle (P value = 0.7). Pathological diagnosis of patients who had major and minor complications are shown in Table 9.

**Table 9:** Pathological diagnosis of patients who had major and minor complications

Biopsy results	Major complications	Minor complications	Total number
Crescentic GN	5	3	8
IgA nephropathy	2	4	6
IFTA	2	1	3
Minimal change	1	4	5
Lupus	1	1	2
Amyloidosis	1	0	1
HUS	1	0	1
Hypertensive nephropathy	1	1	2
Mesangiocapillary GN	0	1	1
Diabetic nephropathy	0	1	1
TIN	0	3	3

*GN* glomerulonephtitis, *IFTA* Interstitial fibrosis, *HUS* haemolytic uraemic syndrome, *TIN* tubulointersitial nephritis

## Discussion

In the present study, we are comparing the safety and adequacy of renal biopsies in ultrasound-assisted and ultrasound-guided renal biopsy techniques. All biopsies were performed by operators of the same level of training in a single centre. Both 14G and 16G needle sizes were used by the operators in this study. Tøndel et al. showed that the rate of complications and inadequacy were higher while using 14G needles than while using 16G needles in a study reviewing 9288 biopsies in the Norwegian kidney biopsy registry from 1988 to 2010 (Tøndel et al. 1988). It also showed that number of passes didn’t have significant effect on serious post-biopsy complications. In this study, groups were compared according to the needle size used, number of passes were not taken as one of the confounders.

In spite of different operators, results were comparable highlighting that the pre biopsy care which is standard in our centre may have impact of the lower and similar complications even with different operators and different needle size. This applies for the similar complication rates and tissue adequacy between hands-free and needle-guided techniques using 16G needle and the slight higher minor complication rates in hands-free technique compared to needle-guided technique using 14G needle.

One of the limitations of the study was that systolic and diastolic blood pressures were not recorded in this study, however, the standard protocol was to exclude any patient with a blood pressure over 160/95 from the procedure. Another limitation is the potential lack of detailed information and data about pre-specified complications in this study. Finally, Post procedure ultrasound was done based on patients symptoms (abdominal pain or macroscopic hematuria) and this may have led to underestimation of complications and under-detection of haematomas.

Baseline creatinine at presentation might be a predictor of risk of complications. Shidham et al. (2005) observed that patients with creatinine level more than 250 mol/L have a higher risk of bleeding.In our study, there was no significant difference between the groups in terms of baseline creatinine.

80.45 % of the biopsies reviewed in this study showed adequate tissue sampling, with no difference between the groups. There is a debate about the number of glomeruli needed for defining adequate tissue sampling. In general, 3–12 glomeruli are needed in each sample to facilitate diagnosis (Oberholzer et al. 1983). It may require one glomerulus to diagnose crescentic glomerulonephritis whereas more than 20 glomeruli may be required to diagnose focal segmental glomerulonephritis. In our study, at least 10 glomeruli were required in each sample to define adequate tissue sampling.

In many studies, the overall complication rates using a spring loaded automated gun ranged from 5 to 18 %. The major complication rates ranged from 0 to 7.7 % and the minor complication rates ranged from 5 to 21 % (Webb et al. 1994; Christensen et al. 1995; Cahen et al. 1995; Song and Cronan 1998; Wang et al. 2015) and this matched the results we observed in our study. It was noticeable that there was no difference between the hands free technique and needle guided technique in terms of major complications.

The most common pathology that major complications occurred with is crescentic glomerulonephritis and this could be explained by the arteritis associated with this type of glomerulonephritis that may increase risk of bleeding.

In conclusion, When using a 14G needle, there is higher risk of minor complications in the free hands (ultrasound-assisted renal biopsy) compared to the needle-guide (ultrasound-guided renal biopsy) technique; no difference in major complications rates was seen between the 2 techniques using a 14G needle. There is no difference in the rates of major or minor complications between free hand and needle-guided technique using 16G needles. Both techniques showed adequate tissue sampling.
