# Erratum to: Post renal biopsy complication rate and diagnostic yield comparing hands free (ultrasound-assisted) and ultrasound-guided biopsy techniques of renal allografts and native kidneys

**DOI:** 10.1186/s40064-016-2577-7

**Published:** 2016-07-04

**Authors:** Hatem Ali, Asam Murtaza, John Anderton, Aimun Ahmed

**Affiliations:** Renal Department, Royal Preston Hospital, Lancashire Teaching Hospitals NHS Foundation Trust, Preston, UK; Nephrology Department, Faculty of Medicine, Ain Shams University, Cairo, Egypt

## Erratum to: Ali et al. SpringerPlus (2015) 4:491 DOI 10.1186/s40064-015-1292-0

The original version of the article (Ali et al. 2015) did not include the second affiliation of Dr. Aimun Ahmed. This has now been included in this erratum.
